# Comparing the Efficacy of Concomitant Therapy with Sequential Therapy as the First-Line Therapy of *Helicobacter pylori* Eradication

**DOI:** 10.1155/2016/1293649

**Published:** 2015-12-28

**Authors:** Sung Min Jung, Dae Young Cheung, Jin Il Kim, Il Kim, Hyeonjin Seong

**Affiliations:** Division of Gastroenterology, Department of Internal Medicine, College of Medicine, The Catholic University of Korea, Seoul KS013, Republic of Korea

## Abstract

*Background*. The decline of *Helicobacter pylori* (*H. pylori*) eradication rates with standard triple therapy resulted in a search for novel therapies for first-line therapy of *H. pylori* infection. *Aim*. The aim of the study is to compare the efficacy of concomitant therapy with sequential therapy as the first-line therapy of *H. pylori* eradication. *Methods*. We reviewed medical records of patients who were confirmed to have *H. pylori* infection and received eradication treatment from September 2012 to March 2015. The concomitant group was treated with rabeprazole, amoxicillin, clarithromycin, and metronidazole for 7 days. The sequential group was treated with rabeprazole and amoxicillin for 5 days and then rabeprazole, clarithromycin, and metronidazole for an additional 5 days. Six weeks after the treatment period, patients in both groups underwent 13C-Urea breath test (UBT) to confirm *H. pylori* eradication. *Results*. The eradication rate was 90.3% in the concomitant group and 85.5% in the sequential group. However, the eradication rates between the two groups showed no statistical difference (*P* = 0.343). *Conclusion*. No statistical difference was found in eradication rates between the two groups. However, in areas where antibiotic resistance is high, concomitant therapy may be more effective than sequential therapy for *H. pylori* eradication.

## 1. Introduction

*Helicobacter pylori (H. pylori)* infection, the major cause of peptic ulcer disease, is a bacterial infection that can lead to inflammation and ulcers in the lining of the stomach and the upper part of the small intestine [1]. It has been proved that curative treatment of* H. pylori* infection markedly reduces relapse of peptic ulcers, bleeding, and gastric cancer [2].

In South Korea, the triple therapy, combination of amoxicillin 2.0 g, proton pump inhibitor (PPI), and clarithromycin 1.0 g or metronidazole 1.5 g, is used as the first-line therapy of* H. pylori* eradication. This therapy was first published in 1998; its guideline was updated for the therapy of* H. pylori* eradication, which was published in 2013 and is still recommended as the first-line therapy. However, with the rising prevalence of antimicrobial resistance, the effectiveness of the standard triple therapy has declined to 62.2–75.0%, which is an unacceptable level worldwide [3, 4]. Because bacterial resistance to clarithromycin is the main cause of this ineffectiveness [5], it is necessary to establish a new regimen for the first-line therapy of* H. pylori* eradication. New regimens containing proton pump inhibitor and three antibiotics have been suggested, among which the concomitant therapy and sequential therapy are the two most popular regimens worldwide.

Concomitant regimen was first introduced in Japan and Germany in 1998 and includes quadruple therapy with standard triple therapy (PPI, clarithromycin, amoxicillin) plus metronidazole. Several studies compared eradication rates of concomitant therapy depending on treatment duration. Although the initial studies from Germany and Japan for concomitant therapy achieved high eradication rates with 5–7 days' treatments [6, 7], a meta-analysis of 15 studies revealed better results with longer treatments (7–10 days versus 3–5 days) [8]. However, a study from Taiwan shortened the duration of concomitant therapy from 10 days to 7 days, while still achieving high eradication rates. This concludes that a 7-day concomitant therapy has a great potential to replace a 7-day triple therapy as the first-line therapy of* H. pylori* eradication [9]. In South Korea, it is standard to use concomitant therapy for 7 days instead of 10 to 14 days, as the eradication rates with 7-day treatments are as high as those with longer duration. Another alternative therapy to the conventional triple therapy is the sequential therapy. Sequential regimen was first introduced in Italy in 2000 and consists of the administration of a PPI and amoxicillin for the first five days followed by a PPI, clarithromycin, and metronidazole for the remaining five days [10, 11].

Recently, several studies have compared the efficacy of the concomitant and sequential therapy. However, because the results are controversial, it is difficult to apply it clinically [1–5, 10–13]. As antibiotics resistance is increasing rapidly, more studies comparing efficacies of the regimens are needed, especially in Asia, where* H. pylori* infection rate is high. Through this study, we compared the eradication rates of concomitant and sequential therapy in a population with established high antibiotics resistance.

## 2. Materials and Methods

### 2.1. Subjects

We reviewed medical records of patients who were confirmed to have gastritis or peptic ulcer with* H. pylori *infection on esophagogastroduodenoscopy and received* H. pylori* eradication treatment from September 2012 to March 2015. Patients who started the eradication therapy in odd numbered days were assigned to concomitant regimen, and patients who started the eradication therapy in even numbered days were assigned to sequential regimen. Six weeks after the treatment period, the patients underwent 13C-Urea breath test (UBT) to confirm* H. pylori* eradication. Among 447 patients who received* H. pylori* eradication treatment from September 2012 to March 2015, we excluded 5 patients who were diagnosed as gastric MALToma or polyps, 15 patients who stopped the eradication treatment by themselves, and 37 patients whose UBT results were not available. As a result, 390 patients were enrolled in this study, 196 patients in concomitant therapy, and 194 patients in sequential therapy.

### 2.2. Methods

#### 2.2.1. *H. pylori* Eradication

196 patients in the concomitant group were treated with rabeprazole 40 mg, amoxicillin 2000 mg, clarithromycin 1000 mg, and metronidazole 1500 mg for 7 days. The other 194 patients in the sequential group were treated with rabeprazole 40 mg and amoxicillin 2000 mg for 5 days and then rabeprazole 40 mg, clarithromycin 1000 mg, and metronidazole 1500 mg for an additional 5 days.

Six weeks after the treatment period, with at least 2 weeks of no administration of PPI, patients underwent UBT to confirm whether the treatment was successful or not. In the case of a treatment failure, a second-line eradication therapy was administered.

### 2.3. Statistical Analysis

The results of this study were analyzed in an intention-to-treat population and a per-protocol population. SAS, version 9.3 for Windows, was used for the statistical analyses, in which the eradication rate was analyzed by the *χ* ^2^ test. *P* values of < 0.05 were deemed statistically significant.

## 3. Results

### 3.1. Patients Characteristics

There was no difference (*P* = 0.732) between the average ages of the groups, which were 56.13 ± 12.01 and 57.27 ± 17.03 years in the concomitant and sequential groups, respectively. The male ratios were 60.2% in concomitant group and 64.4% in sequential group, which also showed no difference (*P* = 0.388). Duodenal ulcers, gastric ulcers, and gastritis were confirmed in 13.2%, 49.5%, and 37.2% in concomitant group, respectively, and in 14.4%, 49.5%, and 36.1% in sequential group, respectively (*P* = 0.936) (Table 1).

### 3.2. Eradication Rate for First-Line Treatment

The successful eradication rate of first-line treatment in the concomitant group was 90.3% (177/196) and 85.6% (166/194) in the sequential group, without statistical difference between the two groups (*P* = 0.3433) (Table 2).

### 3.3. Eradication Rate for Second-Line Treatment

Patients who experienced first-line eradication failure were treated with second-line eradication therapy. They were treated with rabeprazole 40 mg, tetracycline 2000 mg, bismuth 1200 mg, and metronidazole 1500 mg for 14 days. The successful second-line eradication rate in concomitant group was 57.1% (8/14) and 55.0% (11/20) in the sequential group (*P* = 0.9014) (Table 3).

### 3.4. Eradication Rate for Third-Line Treatment

Patients who experienced second-line eradication failure were treated with third-line eradication therapy. They were treated with rabeprazole 40 mg, amoxicillin 2000 mg, and levofloxacin 500 mg for 14 days. The remaining 2 patients in the concomitant group were confirmed to experience successful eradication. However, the remaining 4 patients in the sequential group experienced eradication failure. The eradication rates were 100.0% and 0.0% in concomitant and sequential group, respectively (*P* = 0.0667) (Table 4).

## 4. Discussion

In South Korea,* H. pylori* infection rate is 59.6% in adults, and reinfection rate is 2.9–9.1%, which is higher than in Western countries [3]. Yet* H. pylori *eradication rate following the standard triple therapy has substantially decreased to 62.2–75.0%, which is an unacceptable level worldwide. Therefore, it is necessary to search for novel therapeutic approaches to cure* H. pylori* infections.

Our study showed 90.3% of eradication rate following concomitant therapy, and 85.5% following sequential therapy as first-line therapy of* H. pylori* eradication. The administration of a PPI and 3 antibiotics, whether given concomitantly or sequentially, showed better eradication rate than the standard triple therapy. However, the eradication rate of concomitant and sequential therapy showed no statistical difference (*P* = 0.3433). The eradication rates between the two groups did not show any statistical difference even when we subdivided the data by age, gender, and diagnosis (Tables 5 and 6).

The results of previous studies comparing the efficacy of concomitant therapy with sequential therapy for* H. pylori* eradication were controversial. In a randomized controlled trial, concomitant therapy showed better eradication rate than sequential therapy (intention-to-treat (ITT), 79.4% versus 70.7%, per-protocol (PP), 94.0% versus 84.4%) [12]. Another randomized controlled trial also showed better eradication rate after concomitant therapy, compared to sequential therapy (ITT, 87.0% versus 81.0%, PP, 91.0% versus 86.0%) [14]. However, many other studies showed no difference in eradication rates between concomitant and sequential therapy. In a randomized study, the eradication rates by ITT analysis were 80.8% in concomitant group and 75.6% in sequential group, and the PP analyses were 81.3% and 76.8%, respectively. The eradication rate between the two groups showed no significant difference [15]. A randomized trial of 232* H. pylori*-infected patients from Taiwan also demonstrated similar eradication rates in both therapies [13].

The eradication rates of second-line therapy of concomitant and sequential therapy groups were both very low, 57.1% and 55.0%, respectively. The treatment regimen used in our study was to give rabeprazole 40 mg, tetracycline 2000 mg, bismuth 1200 mg, and metronidazole 1500 mg for 14 days, which is the most commonly prescribed second-line therapy in South Korea. Although the resistance to metronidazole and tetracycline has increased in South Korea, no difference was found in the standard second-line eradication rates [16].

The third-line eradication rates were 100.0% and 0.0% in concomitant and sequential group, respectively. However, there was no statistical difference between the two groups. There are still no guidelines or standard therapies for third-line eradication therapy. In our study, we treated with rabeprazole 40 mg, amoxicillin 2000 mg, and levofloxacin 500 mg for 14 days. We selected levofloxacin because the Korean guidelines recommend levofloxacin for 14 days as a third-line eradication therapy.

Several factors influence the outcome of eradication therapy. A study showed that clarithromycin only and with metronidazole (dual) resistance significantly lowers successful eradication rate after sequential therapy. However, dual resistance did not affect eradication rate after concomitant therapy. The presence of major adverse events was also a predictor of eradication in the sequential group. For concomitant therapy, drug compliance significantly influenced the outcome of treatment efficacy [13]. Because antibiotic resistance is an important factor that influences the outcome of eradication therapy, it is inevitable to get different eradication results in areas where antibiotic resistance rates are different.

South Korea is known to have a high rate of antibiotic resistance. From 2009 to 2012, clarithromycin resistance of* H. pylori *has increased from 7.0% to 16.0%, metronidazole resistance increased from 45.1% to 56.3%, and dual resistance also increased from 16.9% to 23.4% [17, 18]. The reason why the eradication rates are high in spite of the high resistance rates of* H. pylori* in South Korea can be explained by the inconsistency between in vitro and in vivo results. As eradication rates are influenced not only by resistance rates of* H. pylori* to antibiotics, but also by patients' compliance, the discordance between in vitro and in vivo results is inevitable. As a result, although our study revealed no statistical difference in eradication rates after concomitant and sequential therapy, in South Korea, where antibiotic resistance is high, concomitant therapy may be more effective than sequential therapy as the first-line therapy of* H. pylori* eradication.

Our study has two specific strengths. First, we used rabeprazole as the PPI for eradication therapy. Rabeprazole is known to be less influenced by polymorphism of* CYP2C19. *Therefore, by using rabeprazole, we can expect stronger influence of PPI on the efficacy of* H. pylori* eradication compared to other PPIs. Second, this study was completed in a short period of time to minimize antibiotic resistance influenced by other factors and reinfection.

However, our study has also some limitations. First, the number of patients enrolled in the study was not enough to prove statistical difference of eradication rates between the two groups. We needed more than twice or triple the number of enrolled patients to prove the difference. Although our study showed no statistical difference of eradication rates between the two groups, in general, the eradication rate after concomitant therapy appears to be higher in clinical practice. This can be explained by high rate of antibiotic resistance in South Korea, which results in the eradication rate of sequential therapy to be more influenced. Second, only a single method was used to detect* H. pylori* infection. If two different methods were used to test for* H. pylori* infection, more accurate eradication rates could be obtained. To overcome this limitation, biopsies were done at four different gastric mucosae, two at the gastric antrum and two at the gastric body. Also, UBT, which has 98% of accuracy rate, was used to confirm* H. pylori* eradication. Third, the groups were not truly randomized, as the patients were randomized to each group by odd days and even days. However, we think that this limitation was inevitable because the study was retrospective. If the study was conducted prospectively and a better randomization technique was used, it might have yielded better results.

## 5. Conclusions

In conclusion, as the eradication rate of standard triple therapy has declined to an unacceptable level, it is necessary to search for novel therapeutic approaches, especially for the first-line therapy of* H. pylori* eradication. The administration of a PPI and 3 antibiotics, whether given concomitantly or sequentially, showed better eradication rate than the standard triple therapy. Although our study revealed that there is no statistical difference in eradication rates between the two groups, in areas where antibiotic resistance is high, as in South Korea, concomitant therapy may be more effective than sequential therapy for the first-line therapy of* H. pylori* eradication.

## Figures and Tables

**Table 1:** Characteristics of patients *n* (%).

	Concomitant	Sequential	*P* value
	(*n* = 196)	(*n* = 194)	
Age	56.13 ± 12.01	57.27 ± 17.03	0.732
Gender			0.389
Male	118 (60.2)	125 (64.4)	
Female	78 (39.8)	69 (35.6)	
Diagnosis			0.936
Duodenal ulcer	26 (13.3)	28 (14.4)	
Gastric ulcer	97 (49.5)	96 (49.5)	
Gastritis	73 (37.2)	70 (36.1)	

**Table 2:** First-line eradication rates of *Helicobacter pylori*.

	Achieved/analyzed patients (*n*)	Eradication rate	*P* value
Concomitant (*n* = 196)	177/196	90.3%	0.354
Sequential (*n* = 194)	166/194	85.6%	

**Table 3:** Second-line eradication rates of *Helicobacter pylori*.

	Achieved/analyzed patients (*n*)	Eradication rate	*P* value
Concomitant (*n* = 14)	8/14	57.1%	0.977
Sequential (*n* = 20)	11/20	55.0%	

**Table 4:** Third-line eradication rates of *Helicobacter pylori*.

	Achieved/analyzed patients (*n*)	Eradication rate	*P* value
Concomitant (*n* = 2)	2/2	100.0%	0.060
Sequential (*n* = 4)	0/4	0.0%	

**Table 5:** Comparison of eradication rates between men and women in each age group.

	Male			Female		
	Concomitant	Sequential	*P* value	Concomitant	Sequential	*P* value
Age, yr						
<40	80.0%	80.0%	1^*∗*^	100.0%	66.7%	0.455^*∗*^
40~64	95.2%	87.0%	0.068	86.2%	87.2%	0.890
≥65	92.0%	90.9%	1^*∗*^	80.0%	79.2%	1^*∗*^

^*∗*^Fisher exact.

**Table 6:** Comparison of eradication rates among duodenal ulcer, gastric ulcer, and gastritis in each age group.

	Duodenal ulcer			Gastric ulcer			Gastritis		
	Concomitant	Sequential	*P* value	Concomitant	Sequential	*P* value	Concomitant	Sequential	*P* value
Age, yr									
<40	75.0%	100.0%	0.364^*∗*^	87.5%	66.7%	0.577^*∗*^	100.0%	60.0%	0.464^*∗*^
40~64	93.3%	84.2%	0.613^*∗*^	90.6%	87.0%	0.535	91.9%	88.4%	0.737^*∗*^
≥65	100.0%	100.0%	N/A	88.0%	81.8%	0.718^*∗*^	75.0%	90.9%	0.284^*∗*^

^*∗*^Fisher exact.
